# Effects of the resistant starch on glucose, insulin, insulin resistance, and lipid parameters in overweight or obese adults: a systematic review and meta-analysis

**DOI:** 10.1038/s41387-019-0086-9

**Published:** 2019-06-05

**Authors:** Yong Wang, Jing Chen, Ying-Han Song, Rui Zhao, Lin Xia, Yi Chen, Ya-Ping Cui, Zhi-Yong Rao, Yong Zhou, Wen Zhuang, Xiao-Ting Wu

**Affiliations:** 1 Department of Gastrointestinal Surgery, West China Hospital, Sichuan University, Chengdu, China; 2 Healthcare-associated Infection Control Center, Sichuan Academy of Medical Science and Sichuan Provincial People’s Hospital, Chengdu, China; 3 Department of day surgery centre, West China Hospital, Sichuan University, Chengdu, China; 4 Department of Clinical Nutrition, West China Hospital, Sichuan University, Chengdu, China

**Keywords:** Nutrition, Obesity

## Abstract

**Background:**

The role of resistant starch (RS) in glucose, insulin, insulin resistance or sensitivity, and lipid parameters have been reported in several studies and remained controversial. A pooled analysis which assessed these parameters has not been performed. Thus, we conducted a meta-analysis to sum up existing evidence about the issue.

**Methods:**

We searched in MEDLINE and PUBMED for studies that were published before November 2018. Meta-analysis of diabetics and nondiabetics trials were performed by use of a random-effects model.

**Results:**

A total of 13 case–control studies that included 428 subjects with body mass index ≥25 were identified. RS supplementation reduced fasting insulin in overall and stratified (diabetics and nondiabetics trials) analysis (SMD = –0.72; 95% CI: –1.13 to –0.31; SMD = –1.26; 95% CI: –1.66 to –0.86 and SMD = –0.64; 95% CI: –1.10 to –0.18, respectively), and reduced fasting glucose in overall and stratified analysis for diabetic trials (SMD = –0.26; 95% CI: –0.5 to –0.02 and SMD = –0.28; 95% CI: –0.54 to –0.01, respectively). RS supplementation increased HOMA-S% (SMD = 1.19; 95% CI: 0.59–1.78) and reduced HOMA-B (SMD =–1.2; 95% CI: –1.64 to –0.77), LDL-c concentration (SMD =–0.35; 95% CI: –0.61 to −0.09), and HbA1c (SMD = –0.43; 95% CI: –0.74 to –0.13) in overall analysis.

**Conclusions:**

This meta-analysis has provided evidence that RS supplementation can improve fasting glucose, fasting insulin, insulin resistance and sensitivity, especially for diabetic with overweight or obesity. However, owing to potential sophistication, individual difference and composition of intestinal microbiota, this result should be carefully taken into account.

## Introduction

Overweight and obesity have been a worldwide epidemic and led to a rise in the insulin resistance-related morbidities, progression to type 2 diabetes and increasing risk of cardiovascular disease^1,2^. It is difficult to achieve or maintain weight loss for many people and we have proposed dietary strategies based on reducing the absorptivity or amount of glucose in the diet to improve metabolic health, rather than depending on weight loss^3^. Resistant starch, as a dietary ingredient, can slow digestion, reduce abdominal fat^4–6^ and cholesterol^7^ in rodents and human. RS increases systemic insulin sensitivity and significantly reduces adipose tissue decomposition, which has clinical significance in the care and prevention of diabetes^8^. Although an association between RS supplementation and insulin concentrations, insulin sensitivity, and lipid parameters is biologically credible, the results of epidemiological studies on this relationship are inconsistent.

Many studies from different countries have been published to report the effects of RS about glucose, insulin, insulin resistance and sensitivity, and lipid parameters, however, no systematic analysis on this issue is still reported so far. Therefore, a meta-analysis was performed to sum up the existing evidence about this topic.

## Methods

### Search strategy

We performed a search of PubMed and Medline databases. The final search was conducted in October 2018 and combinations of search terms were included (resistant starch or RS) and (blood glucose or plasma insulin or insulin resistance or insulin sensitivity or cholesterol or triglyceride or LDL or HDL or hyperlipidemia or triacylglycerol or dyslipidemia) and (overweight or obesity). The reference lists of each papers were scanned by us to identify additional studies. If necessary, we try to contact the author for more information.

### Selection criteria

Studies were included if they met the following criteria, which included clinical trials; controlled; Intervention of obesity or overweight (BMI ≥ 25) with resistant starch; with adults (>18 years old); baseline characteristic without difference; without acute effect of RS; assessing fasting glucose or fasting insulin or plasma lipid or insulin sensitivity or insulin resistance as outcomes; with data of the related outcomes or data necessary to calculate them. For potentially qualified articles that are with unclear information, we contacted the correspondence author via email and asked for more explanations. The articles were included only if the problem has been solved and met the selection criteria. No duplicate or triplicate clauses are included.

### Data extraction

All data were extracted independently and cross-checked by three reviewers (Y.W., J.C., and X.T.W.) according to the selection criteria. Articles would be discussed again in case of divergent opinions. The following information were extracted: patient characteristics (gender, age, and BMI), sample size, resistant starch or placebo components, dosage, duration of treatment and result (mean and standard deviation after supplement). Outcomes included plasma lipid (total cholesterol, low-density lipoprotein cholesterol (LDL-c), high-density lipoprotein cholesterol (HDL-c), and triglycerides), insulin sensitivity, insulin resistance, B-cell function, fasting insulin and glucose. For studies that do not give the average and standard deviation values of any relevant results, we contacted the correspondence authors to require these values, and we included the articles that can offer these data.

### Quality assessment

Quality assessment was performed according to the quality assessment toll for quantitative studies, Effect public health practice project (EPHPP)^9^. The EPHPP toll include six evaluation criteria: selection bias, study design, confounding factors, blind method, data collection methods and withdrawals, and dropouts. According to the characteristics of each criterion reported in the study, the six criteria were rated as “strong”, “moderate” or “weak”. Once the standard scores are aggregated, each study will receive an overall assessment of strong, moderate or weak quality. In order for a study to be rated as “strong”, four of the six quality assessment criteria must be rated as strong without weak ratings. if less than four criteria were rated as strong and one criterion was as weak, it achieved a rating of “moderate”^9^.

### Statistical analysis

We performed all statistical analyses with Statistical Software-STATA, version 12.0. Mean differences (MD) between intervention (RS) and control group for each of the above results were summarized using the random-effect model, which was applied to the meta-analyses when the studies were clinically heterogeneous. The values of mean change from baseline standard deviations were used to calculate missing standard deviations. When some trials report the low and high end or 25th to 75th percentiles of the range, the standard deviation was regarded as the formula range/4^10^.

Studies with resistant starch were divided into two groups (nondiabetic and diabetic), because of different composition of gut microbiota between the two populations^11,12^, and due to high concentrations of insulin and glucose in the diabetic population, which may produce more significant results through interventions. We used the *Q* and *I*^2^ statistics to test statistical heterogeneity among studies^13^. we considered *P* value of less than 0.1 as a statistically significant heterogeneity for the *Q* statistic. If a study has a heterogeneous source, it was excluded of the analysis. Data synthesis of these heterogeneous studies was presented in a narrative analysis. the Egger weighted regression method was used to assess publication bias^14^; which considered *P* value of less than 0.1 as a statistically significant publication bias.

## Results

### Search results

**Fig. 1:**
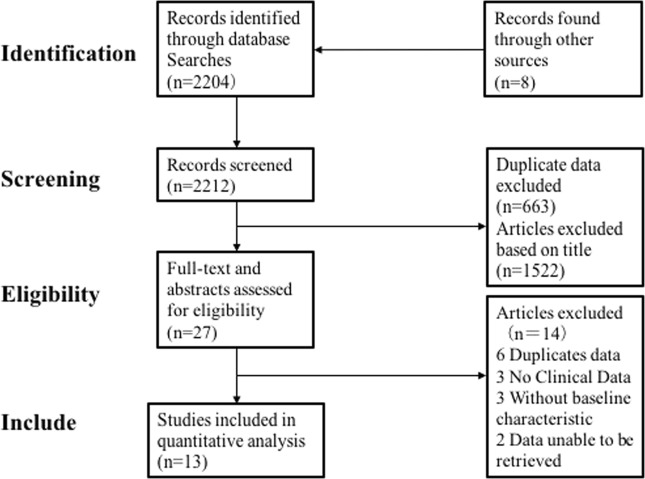
Screening and selection process of studies

There were 2212 articles identified in the search, the titles and abstracts of the articles were screened. Only 27 articles were considered eligible. After review of full text articles, 13/27 met the inclusion and were eligible in this meta-analysis. Figure 1 showed the selection process.

### Baseline characteristics

The thirteen included studies^15–27^ were published between 2004 and 2018. The Table 1 showed the characteristics of these studies. Of all the studies. three of them were from Europe^16,19,24^; seven from America^15,17,18,22,23,25,27^; two from Middle East^20,21^; and one from Asia^26^. Of the thirteen trials, five of them were randomized, crossover study, the other eight were randomized controlled trials. Sample sizes were 12–60 cases and follow-up ranged from 2 to 12 weeks. The doses of RS ranged from 10 to 45 g per day. The effect of taking resistant starch versus placebo on glycemic status, insulin and lipid profile are described in Table 2. There are three studies^18,23,27^, including four or two groups, respectively in their analysis and all were included for the meta-analysis. There was only one study in diabetic or nondiabetic group for some parameters such as HOMA%B, HbA1c, HOMA%S, HOMA-IR, and LDL-c so we did not make stratified analysis for these parameters.

**Table 1:** Characteristics of included studies

Study (year)	Country	Supplement	Study design	Population	Sex		BMI placebo vs. control		Age (years) placebo vs. control	Sample size	Intervention (RS dose, g)	Control (RS dose, g)	Duration	Results
Park OJ (2004)	Korea	RS	Placebo-control study	Overweight and obese subjects		F		26.6 ± 0.7 vs. 27.9 ± 0.5	42.3 ± 3.1 vs. 43.6 ± 2.8	25	Resistant starch (40)	Corn starch (0)	3 weeks	↓fasting glucose ↓fasting insulin ↓TC ↑TG ↓HDL-c ↓LDL-c
Castillo JL (2010)	Mexico	NBS	Crossover study	Obese adults with type 2 diabete		M F		34.89 ± 2.32	51.7 ± 5.6	30	NBS (24)	Soy milk (0)	4 weeks	↓Fasting glycemia ↓Fasting insulin ↑HOMA-IR ↓TC ↓HDL−c ↑ TG ↓HbA1c
Johnston KL **(**2010**)**	UK	RS	Placebo-control study	Adults with metabolic syndrome		M F		30.4 ± 1.15 vs. 31.3 ± 1.7	50.1 ± 4.05 vs. 45.2 ± 3.55	20	RS and RDS (40)	RDS (0)	12 weeks	↓HOMA %S ↓HOMA %B ↓Insulin sensitivity
Bodinham CL (2012)	UK	RS	Crossover study	Overweight individuals		M F		28.2 ± 0.4	37 ± 4.0	12	RS and RDS (40)	RDS (0)	4 weeks	↓Fasting glucose ↑Fasting insulin ↑Fasting TG ⇿TC
Maki KC (2012)	USA	RS	Crossover study	Healthy adults		M F		30.6 ± 0.5	49.5 ± 1.6	33	Corn starch containing 60% RS (30) corn starch containing 60% RS (15)	Control starch containing no RS (0) control starch containing no RS (0)	4 weeks	Male: ⇿Fasting glucose ↓Fasting insulin ⇿HOMA%B ⇿HOMA%S Female: ⇿Fasting glucose ↓Fasting insulin ↓HOMA%B ⇿HOMA%S Male: ⇿Fasting glucose ↓Fasting insulin ↓HOMA%B ⇿HOMA%S Female: ⇿Fasting glucose ↓Fasting insulin ↓HOMA%B ⇿HOMA%S
Robertson MD (2012)	France	RS	Crossover study	Healthy subjects with insulin resistance		M F		33.8 ± 1.9	48.9 ± 3.9	15	RS and RDS (40)	RDS (0)	8 weeks	↓Fasting glucose ↓Fasting insulin ↓HOMA-%B ↓HOMA-IR ↓Fasting TC ↑Fasting TG
Gargari BP (2015)	Iran	RS	Placebo-control study	Adults with type 2 diabetes		F		30.8 ± 5.2 vs 31.5 ± 4.5	49.6 ± 8.4 vs 49.5 ± 8.0	60	RS2 (10)	Maltodextri-n (0)	8 weeks	↓Fasting plasma glucose ↓TG ↓TC ↓HDL-c ↓LDL-c ↓HbA1c
Karimi P (2015)	Iran	RS	Placebo-control study	Adults with type 2 diabetes		F		31 ± 4.9 vs 31.5 ± 4.5	48.6 ± 7.9 vs 49.5 ± 8.0	56	RS2 (10)	Maltodextri-n (0)	8 weeks	↓Fasting glucose ↓Fasting insulin ↓HOMA-IR ↓HbA1c
Dainty SA (2016)	Canada	RS	Crossover study	Adults with risk of Type 2 Diabetes		M F		30.2 ± 0.57	55.3 ± 1.59	24	RS bagel (25)	Control bagel (0)	8 weeks	↓Fasting plasma glucose ↓Fasting serum insulin ↓HOMA-IR ↓HOMA%B ↑HOMA%S
Bergeron N (2016)	USA	RS	Crossover study	Men and post-menopausal women		M F		31 ± 2	44 ± 14	52	Higher-CHO study: RS (66) Lower-CHO study: RS (48)	Higher-CHO study: RS (4) Lower-CHO study: RS (3)	2 weeks	Higher-CHO study: ↓Fasting glucose ↓Fasting insulin ↓TC ⇿TG ⇿HDL-c ⇿LDL-c Lower-CHO study: ↑Fasting glucose ↑Fasting insulin ⇿TC ⇿TG ⇿HDL-c ⇿LDL-c
Gower BA (2016)	USA	RS	Crossover study	Nondiabetic women		F		29.8 ± 6.7	48.3 ± 12.6	23	High-amylose Maize(RS) (19.05) high-amylose Maize(RS) (11.35)	Control starch containing RS (3.18) control starch containing RS (3.18)	4 weeks	Insulin sensitive: ↑Fasting glucose ↑Fasting insulin ↑TC ↑TG ⇿HDL-c Insulin resistance: ↓Fasting glucose ↓Fasting insulin ↑TC ↑TG ↓HDL-c Insulin sensitive: ⇿Fasting glucose ↑Fasting insulin ↑TC ↓TG ⇿HDL-c Insulin resistance: ↓Fasting glucose ↓Fasting insulin ↓TC ⇿TG ⇿HDL-c
Schioldan AG (2017)	Denmark	RS	Crossover study	Participants with metabolic syndrome		M F		>25	58 ± 11	19	HCD: RS (21)	WSD: RS (3)	4 weeks	⇿glucose ↓insulin ↓TG ↓TC ⇿HDL-c ↓LDL-c ↑HOMA-IR
Peterson CM (2018)	American	RS	Placebo-control study	Adults with prediabetes		M F		54 ± 10 vs. 55 ± 10	35.5 ± 4.04 vs. 35.7 ± 5.2	59	High-amylose maize (RS) (45)	Amioca cornstarch (0)	12 weeks	⇿fasting glucose ↑fasting insulin ↓TC ↓TG ↓HDL-c ↓LDL-c ↓HbA1c

*BMI* body mass index, *M* male, *F* female, *RS* resistant starch, *RDS* rapidly digestible starch, *NBS* native banana starch, *IFG* impaired fasting glucose, *IGT* impaired glucose tolerance, *NSP* nonstarch polysaccharide, *CHO* carbohydrate, *WSD* refined carbohydrates, *HCD* healthy carbohydrate diet, *MID* mid-age adults, *ELD* elderly adults, *HbA1c* glycated hemoglobin

*HOMA %S* fasted oral insulin sensitivity, assessed by homeostasis model assessment, *HOMA %B* b-cell function, assessed by homeostasis model assessment, *HOMA-IR* insulin resistance index, assessed by homeostatic model assessment, *TG* triglyceride, *TC* total cholesterol, *HDL-c* high density lipoprotein cholesterol, *LDL-c* low density lipoprotein cholesterol, *NR* not report

⇿ no significant difference between the intervention and control groups after intervention

↓ significantly lower than control group after intervention

↑ significantly higher than control group after intervention

**Table 2:** Impact of consuming resistant starch versus placebo on glycemic status, insulin, and lipid profile at the end of study

Study (year)	Fasting glucose (momol/L) placebo vs. control	Fasting insulin (mIU/L) placebo vs. control	HOMA %B placebo vs. control	HOMA %S placebo vs. control	HOMA-IR placebo vs. control	HbA1c (%) placebo vs. control	TC (mg/dL) placebo vs. control	TG (mg/dL) placebo vs. control	HDL-c (mg/dL) placebo vs. control	LDL-c (mg/dL) placebo vs. control
Park OJ (2004)	5.33 ± 0.22 vs. 5.33 ± 0.33	17.21 ± 4.95 vs. 33.57 ± 13.93	NR	NR	NR	NR	123.74 ± 7.73 vs. 123.52 ± 7.73	141.6 ± 56.64 vs. 127.44 ± 49.56	33.64 ± 2.32 vs. 32.1 ± 2.32	107.89 ± 5.41 vs. 110.6 ± 5.41
Castillo JL (2010)	8.0 ± 1.15 vs. 8.16 ± 0.49	11.2 ± 1.4 vs. 13 ± 1.23	NR	NR	NR	6.3 ± 0.21 vs. 6.3 ± 0.25	206 ± 8.13 vs. 207.5 ± 7.25	252 ± 119.2 vs. 187 ± 23	42.07 ± 1.5 vs. 44.07 ± 2.75	NR
Johnston KL (2010)	NR	NR	162 ± 12.7 vs. 176 ± 24.2	80.2 ± 12.7 vs. 70.1 ± 5.68	NR	NR	NR	NR	NR	NR
Bodinham CL (2012)	4.8 ± 0.1 vs. 5.1 ± 0.1	88.6 ± 9.5 vs. 85.4 ± 7.8	NR	NR	NR	NR	185.62 ± 11.6 vs. 185.62 ± 11.6	141.6 ± 26.55 vs. 115.05 ± 17.7	NR	NR
Maki KC (2012)	30 g RS: male 1.8 ± 0.22 vs. 1.7 ± 0.1 female 5.4 ± 0.1 vs. 5.5 ± 0.1 15 g RS: male 1.8 ± 0.8 vs. 1.7 ± 0.1 female 5.5 ± 0.1 vs. 5.5 ± 0.1	30 g RS: male 58.5 ± 4.7 vs. 62.5 ± 4.7 female 47.5 ± 4.9 vs. 56.2 ± 4.9 15 g RS: male 50.1 ± 4.7 vs. 62.5 ± 4.7 female 51.6 ± 5 vs. 56.2 ± 4.9	30 g RS: male 70.3 ± 6.1 vs. 78.1 ± 6.1 female 80.7 ± 6.1 vs. 89.3 ± 6.1 15 g RS: male 61.5 ± 6.1 vs. 78.1 ± 6.1 female 84.2 ± 6.2 vs. 89.3 ± 6.1	30 g RS: male 4.6 ± 0.1 vs. 4.6 ± 0.1 female 4.7 ± 0.1 vs. 4.5 ± 0.1 15 g RS: male 4.7 ± 0.1 vs. 4.6 ± 0.1 female 4.6 ± 0.1 vs. 4.5 ± 0.1	NR	NR	NR	NR	NR	NR
Robertson MD (2012)	5 ± 0.1 vs. 5.2 ± 0.11	108 ± 8.4 vs. 129 ± 10.2	175.9 ± 11.9 vs. 182.5 ± 12.6	NR	2.5 ± 0.2 vs. 2.9 ± 0.2	NR	162.41 ± 15.47 vs. 166.28 ± 11.6	123.9 ± 17.7 vs. 106.5 ± 8.85	NR	NR
Gargari BP (2015)	8.44 ± 2.02 vs. 8.67 ± 0.79	NR	NR	NR	NR	7.7 ± 1.3 vs. 8.3 ± 1	181.5 ± 39.1 vs. 203.1 ± 45.6	146.5 ± 63.7 vs. 216.7 ± 59.8	45.2 ± 9.5 vs. 38.2 ± 7.1	101.7 ± 40.8 vs. 119.1 ± 41.2
Karimi Pv (2015)	8.44 ± 2.02 vs. 8.86 ± 0.79	69.86 ± 12.61 vs. 98.9 ± 32.7	NR	NR	3.76 ± 1.7 vs. 5.6 ± 2.5	7.7 ± 1.15 vs. 8.5 ± 1.15	NR	NR	NR	NR
Dainty SA (2016)	5.29 ± 0.075 vs. 5.31 ± 0.075	68.7 ± 5.63 vs. 88.2 ± 7.08	140 ± 18 vs. 170 ± 22	39 ± 6 vs. 29 ± 4	2.57 ± 1.1 vs. 3.43 ± 1.3	NR	NR	NR	NR	NR
Bergeron N (2016)	66 g RS 5.26 ± 0.55 vs. 5.3 ± 0.55 48 g RS 5.38 ± 0.51 vs. 5.27 ± 0.49	66 g RS 59.9 ± 31.34 vs. 67.56 ± 38.3 48 g RS 61.29 ± 28.56 vs. 55.72 ± 27.16	NR	NR	NR	NR	66 g RS 166.28 ± 27.46 168.21 ± 30.16 vs. 48 g RS 164.35 ± 24.36 vs. 164.73 ± 25.14	66 g RS 108.86 ± 38.06 vs. 109.74 ± 38.94 48 g RS 95.58 ± 45.14 vs. 100 ± 65.49	66 g RS 41.76 ± 6.57 vs. 42.92 ± 8.12 48 g RS 41.38 ± 8.12 vs. 41.76 ± 8.12	66 g RS 102.86 ± 18.56 vs. 103.25 ± 20.88 48 g RS 103.64 ± 19.34 vs. 104.02 ± 20.88
Gower BA (2016)	19.05 g RS insulin sensitive: 5.46 ± 0.72 vs. 5.56 ± 0.68 insulin resistance 5.46 ± 0.52 vs. 5.56 ± 0.68 11.35 g RS Insulin sensitive: 5.09 ± 0.47 vs. 4.88 ± 0.25 Insulin resistance 4.83 ± 0.37 vs. 4.88 ± 0.25	19.05 g RS insulin sensitive: 66.86 ± 48.06 vs. 72.44 ± 32.04 insulin resistance: 68.26 ± 32.04 vs. 72.44 ± 32.04 11.35 g RS insulin sensitive: 32.04 ± 8.36 vs. 27.86 ± 12.54 Insulin resistance: 32.74 ± 8.36 vs. 27.86 ± 12.54	NR	NR	NR	NR	19.05 g RS insulin sensitive: 190.4 ± 37.4 vs. 187.6 ± 38.3 insulin resistance: 181.2 ± 24.4 vs. 187.6 ± 38.3 11.35 g RS insulin sensitive: 190.8 ± 41.9 vs. 179.6 ± 32.2 insulin resistance: 182.4 ± 34.4 vs. 179.6 ± 32.2	19.05 g RS insulin sensitive: 118.2 ± 59.1 vs. 117 ± 47.9 insulin resistance: 111.8 ± 59.1 vs. 117 ± 47.9 11.35 g RS insulin sensitive: 96.1 ± 31.1 vs. 83.7 ± 19.6 insulin resistance: 79.1 ± 27 vs. 83.7 ± 19.6	19.05 g RS insulin sensitive: 56.9 ± 14.3 vs. 59 ± 18 insulin resistance: 58.8 ± 9.6 vs. 59 ± 18 11.35 g RS insulin sensitive: 62.9 ± 11.9 vs. 61.9 ± 8.1 insulin resistance: 62.7 ± 8.9 vs. 61.9 ± 8.1	NR
Schioldan AG (2017)	5.9 ± 0.6 vs. 5.9 ± 0.6	73.1 ± 17.55 vs. 90.8 ± 14.3	NR	NR	3.88 ± 0.6 vs. 3.61 ± 0.5	NR	176.34 ± 29.39 vs. 183.68 ± 33.64	138.06 ± 21.46 vs. 139.83 ± 19.69	39.44 ± 10.44 vs. 39.83 ± 9.67	104.41 ± 28.62 vs. 110.21 ± 30.55
Peterson CM (2018)	6 ± 0.44 vs. 6.11 ± 0.44	22.29 ± 5.4 vs. 21.29 ± 6.3	NR	NR	NR	5.7 ± 0.2 vs. 5.8 ± 0.2	181.67 ± 29 vs. 184.67 ± 17.72	108.25 ± 48 vs. 100.25 ± 25.6	114.14 ± 12 vs. 110.14 ± 24	45.9 ± 4.9 vs. 48.7 ± 4.08

*RS* resistant starch, *HOMA %S* fasted oral insulin sensitivity, assessed by homeostasis model assessment, *HOMA %B* b-cell function, assessed by homeostasis model assessment, *HOMA-IR* insulin resistance index, assessed by homeostatic model assessment, *HbA1c* glycated hemoglobin, *TG* triglyceride, *TC* total cholesterol, *HDL-c* high-density lipoprotein cholesterol, *LDL-c* low-density lipoprotein cholesterol, *NR* not report

### Quality assessment

Twelve studies were rated as strong^15–23,25–27^ and one study was as moderate^24^ through the EPHPP method. All the studies were rated as strong according to the criteria “selection bias”, “study design”, “confounders”, “withdrawals and dropouts”, and “data collection methods”, while one study^24^ was evaluated as weak in the criteria of “blinding”.

### Overall and stratified analysis

We performed the meta-analyses on twelve studies^15,17–27^ for fasting glucose; ten trials^15,17–19,21,23–27^ for fasting insulin; eight trials for total cholesterol^15,17,19,20,23–25,27^ and triglycerides^15,17,19,23–27^; four trials for HOMA-IR^15,21,22,24^ and seven trials for HDL-c^15,20,23–27^; three trials^16,18,22^ for HOMA-S% and HOMA-B%, and five trials for LDL-c^20,23–26^. Three studies^18,23,27^, included two groups, respectively, in their analysis and this meta-analysis included all the groups. One data were removed from analysis of the insulin and total cholesterol respectively because of a heterogeneous source as was observed through inspecting of the forest plots and that does not affect the outcome of overall analysis.

**Fig. 2:**
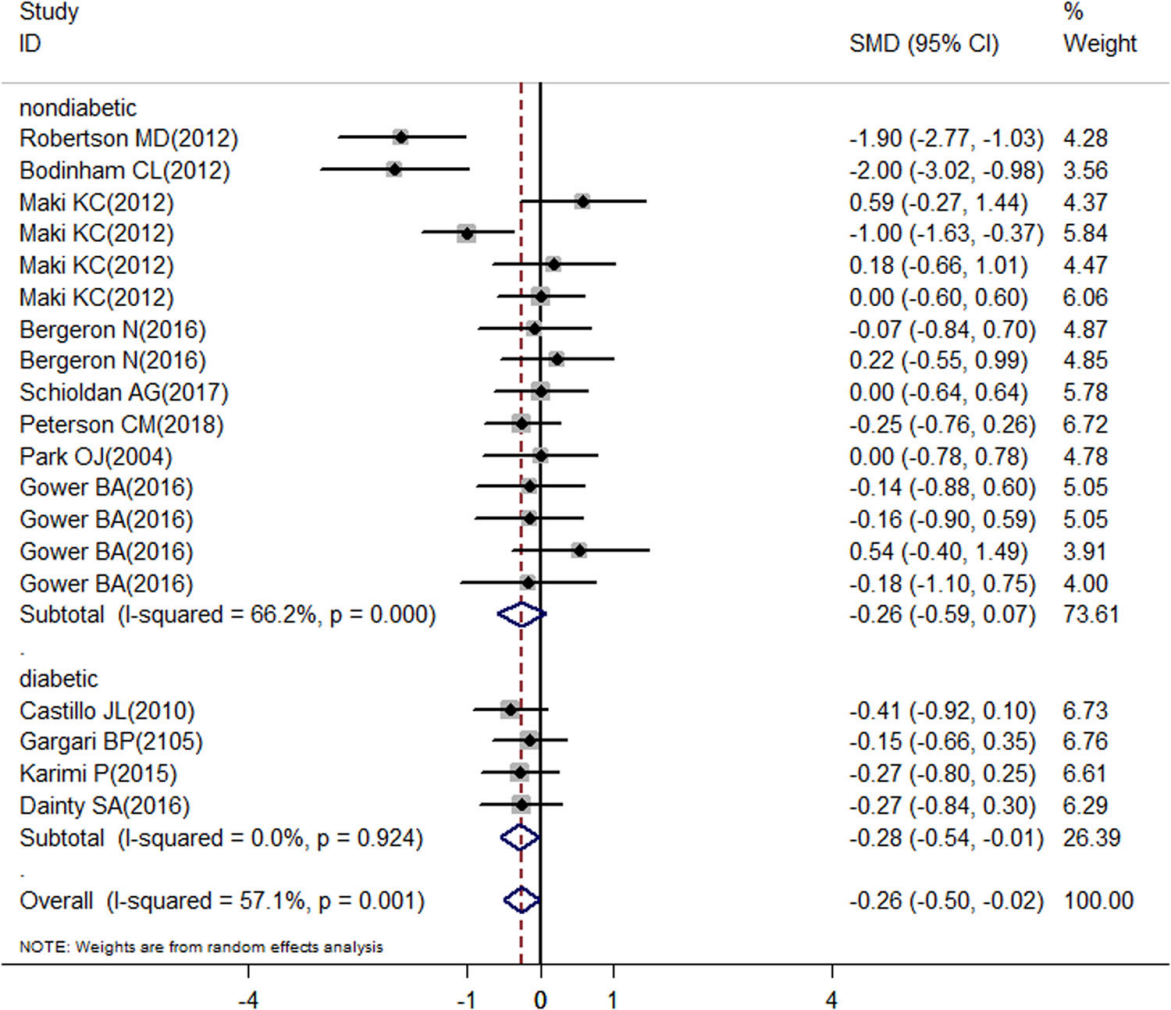
Forest plot for resistant starch and control groups in fasting glucose

**Fig. 3:**
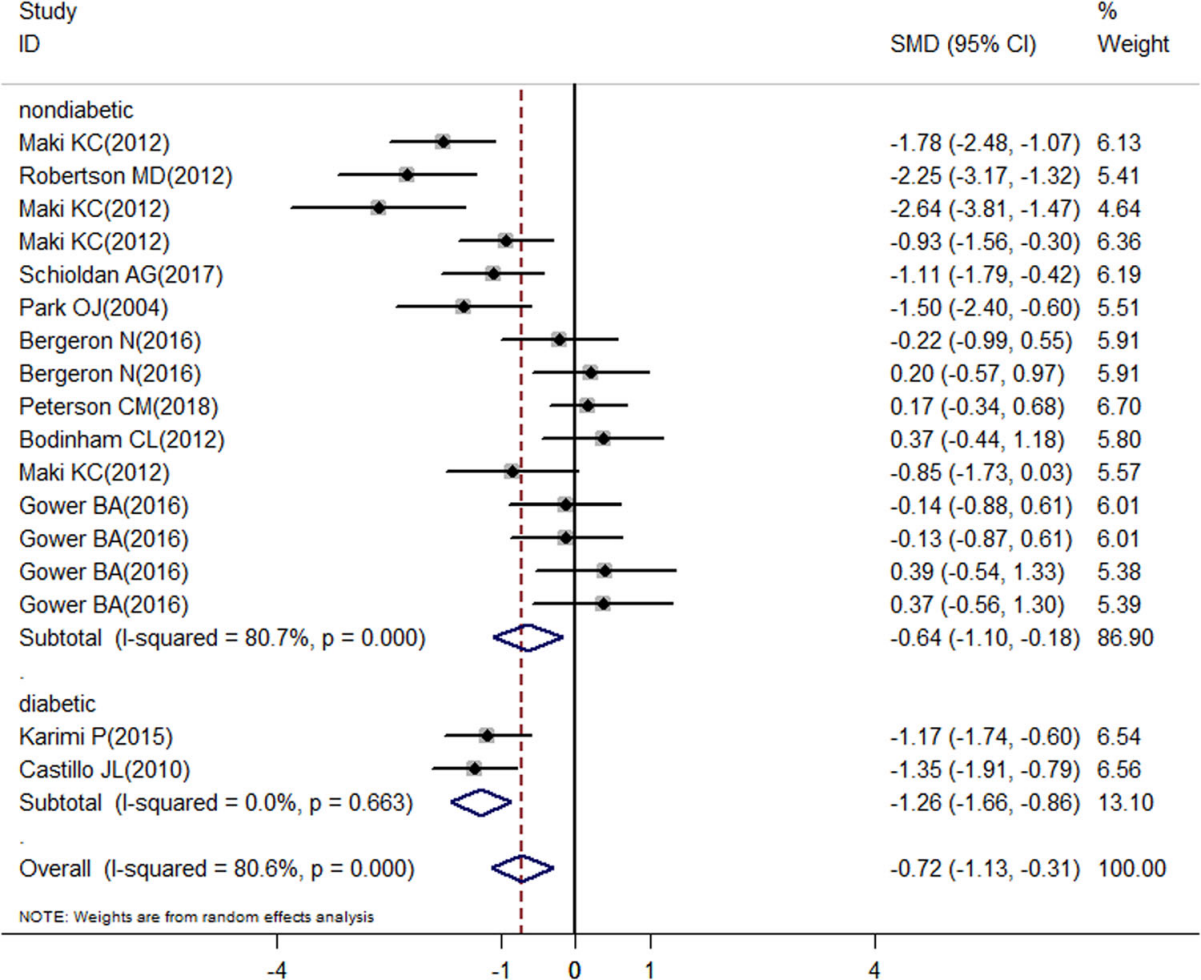
Forest plot for resistant starch and control groups in fasting insulin

**Fig. 4:**
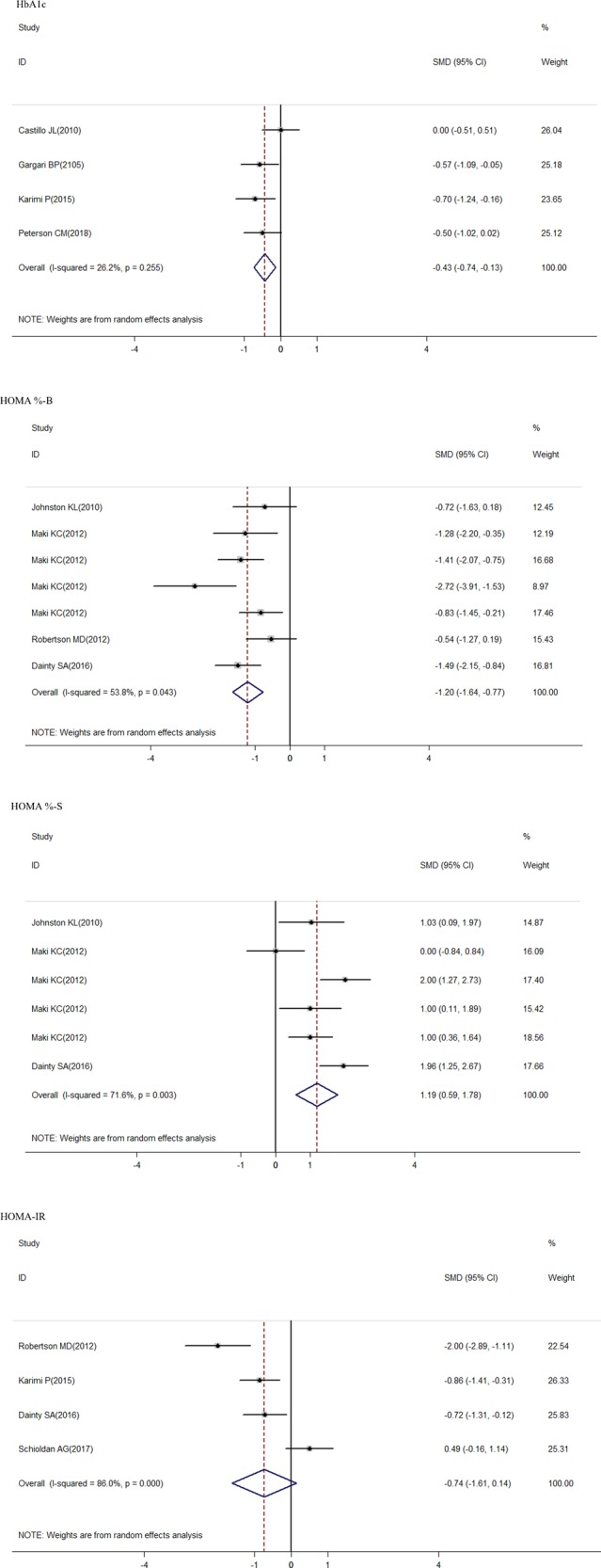
Forest plot for resistant starch and control groups in HOMA-S%, HOMA-B%, HOMA-IR, and HbA1c

**Fig. 5:**
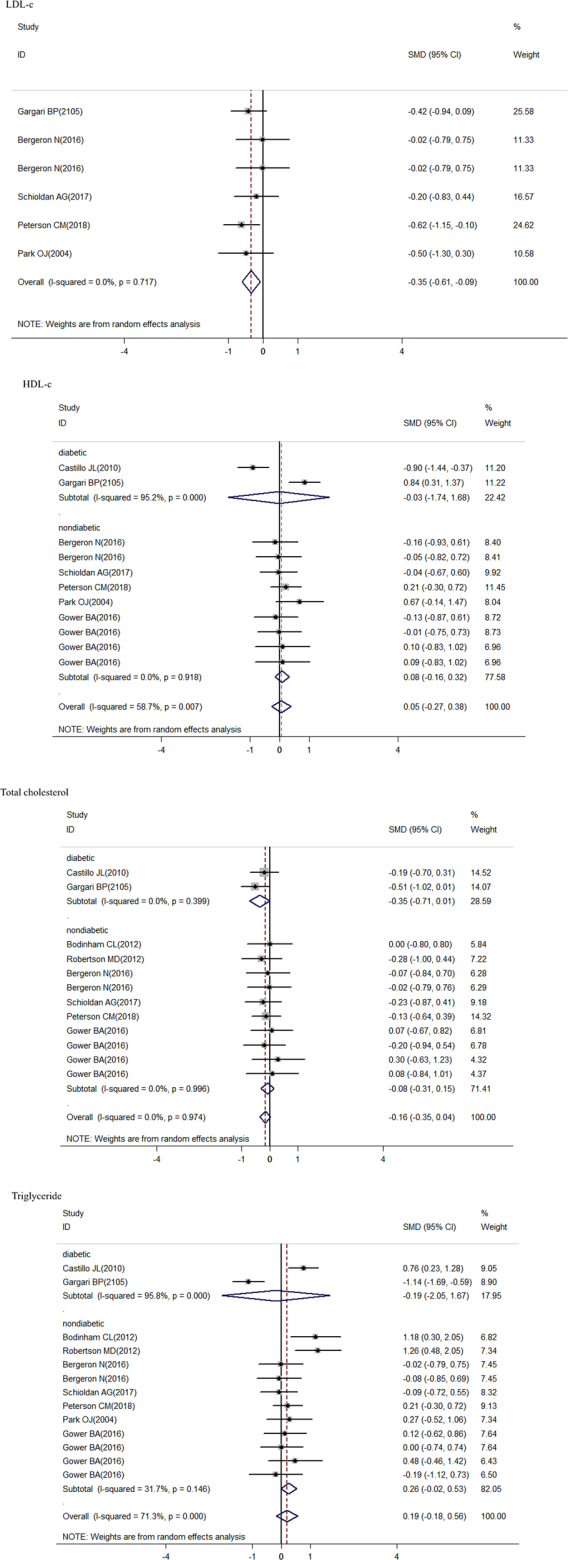
Forest plot for resistant starch and control groups in total cholesterol, LDL-c, HDL-c, and triglycerides

The overall meta-analysis showed a significant decrease in the fasting glucose after RS consumption (SMD = –0.26; 95% CI: –0.5 to –0.02; *P* = 0.035) (Fig. 2); in the fasting insulin concentration (SMD = –0.72; 95% CI: –1.13 to –0.31; *P* = 0.001) (Fig. 3); in the LDL-c concentration (SMD = –0.35; 95% CI: –0.61 to –0.09; *P* = 0.008) (Fig. 5); in the HOMA-B% (SMD = –1.2; 95% CI: –1.64 to –0.77; *P* = 0.000) and in the HbA1c (SMD = –0.43; 95% CI: –0.74 to –0.13; *P* = 0.005), but there was a significant increase in the HOMA-S% (SMD = 1.19; 95% CI: 0.59–1.78; *P* = 0.000) (Fig. 4). Nonsignificant effect was showed in HDL-c; total cholesterol; triglycerides concentration, and HOMA-IR (SMD = 0.05; 95% CI: –0.27–0.38; *P* = 0.759; SMD = 0.21; 95% CI: –0.35–0.04; *P* = 0.113; SMD = 0.19; 95% CI: –0.18–0.56; *P* = 0.758 and SMD = –0.74; 95% CI: –1.61 to 0.14; *P* = 0.098; respectively) (Figs. 4 and 5).

There was a significant decrease in the fasting insulin showed in the diabetic and nondiabetic subgroups (SMD = –1.26; 95% CI: –1.66 to –0.86; *P* = 0.000; SMD = –0.64; 95% CI: –1.10 to –0.18; *P* = 0.006, respectively) (Fig. 3). A significant decrease in fasting glucose was showed by studies with diabetics (SMD = –0.28; 95% CI: –0.54 to –0.01; *P* = 0.04) (Fig. 2). Both nondiabetic and diabetic subgroups indicated a non-significant effect in HDL-c; total cholesterol; and triglycerides concentration (Fig. 5).

There was significant heterogeneity in the analysis of fasting glucose, fasting insulin, HDL-c, triglycerides, HOMA-S%, HOMA-B%, and HOMA-IR (*I*^2^ = 57.1%, 80.6%, 58.7%, 71.3%, 71.6%, 53.8%, and 86%, respectively). The heterogeneity in the analysis for fasting glucose, fasting insulin due to the trials with nondiabetics (*I*^2^ = 66.2%, 80.7%, respectively). However, the heterogeneity for HDL-c and triglycerides due to the trial with diabetics and low study (*I*^2^ = 95.2%, 95.8%, respectively), we did not make stratified analysis for HOMA-S%, HOMA-B%, and HOMA-IR because of the few data.

### Adverse effects

Adverse effects after RS supplementation were reported in five studies, including flatulence^15,23,25^, abdominal discomfort^18,23–25^, diarrhea and swelling^25^, fullness^18,23,25^, nausea, and constipation^18,25^. Most of which were mild and disappeared after few days of consumption. Three studies^16,21,26^ reported no adverse reaction after RS supplementation and five ones^17,19,20,22,27^ did not report adverse effects as a result.

### Publication bias

Using the Egger weighted regression method, there was no publication bias found in analysis for fasting glucose (*P* = 0.445), fasting insulin (*P* = 0.245), total cholesterol (*P* = 0.182), HDL-c (*P* = 0.894), HOMA-S% (*P* = 0.476), HOMA-B% (*P* = 0.314), HOMA-IR (*P* = 0.573), LDL-c (*P* = 0.153), and triglycerides (*P* = 0.379).

## Discussion

In this meta-analysis of 13 studies involving 428 subjects, we saw that RS had an increasing effect on HOMA-S% and a lowering effect on fasting glucose, fasting insulin, LDL-c concentration, HbA1c, and HOMA-B% were found in overweight or obese adults. In our study, there was no significant effect of RS supplementation on HDL-c, total cholesterol, triglycerides, and HOMA-IR, which was in line with another study^28^. Meanwhile, the meta-analysis of the prebiotics showed that the inulin could reduce the total cholesterol, LDL-c and triglycerides concentrations in patients with hyperlipidemia^29^.

In our study, there were 6–13 data for analysis of total cholesterol, HDL-c, LDL-c, and triglycerides. A mild decrease was showed in the trials for analysis of total cholesterol^15,19,20,23–26^, HDL-c^15,20,25,26^, LDL-c^20,24–26^, and triglycerides^20,24,26^. There was a mild increase showed in the trial for analysis of total cholesterol and triglycerides^27^, and no significant difference after RS supplementation was found in the trials for analysis of total cholesterol^23^, HDL-c^23,24,27^, LDL-c^23^, and triglycerides^23,24^, which could explain the lack of significant impact in the analyses. Four of six data reported a slight decrease in the LDL-c as a result of significant effect in the nondiabetic subgroup and overall analyses of LDL-c. Meanwhile, a meta-analysis reported a significant reduce in total cholesterol and LDL-c after the prebiotics supplementation in overweight or obese adults^30^. Previous studies have shown that different types of RS have opposite effects on glucose and lipid levels in healthy subjects and T2DM patients. The diversity of results may be due to differences in diet composition, dietary RS content, source of RS, dosage and type of RS, and the pathological status of the patients which can be a cause in significant heterogeneity in analysis. However, low-sample size may be the most likely reason.

Four studies were for analysis of HOMA-IR^19,21,22,24^ and HOMA-B%^16,18,19,22^, three for HOMA-S%^16,18,22^. A mild decrease was showed in the data for analysis of HOMA-IR^19,21,24^; HOMA-B%^16,18,19,22^ and HOMA-S%^16^. An increase^22^ was showed for analysis of HOMA-S%, which can explain the effect in the overall analysis. Recent studies from animal models containing HAM-RS2 have shown an increase in pancreatic beta cell^31^. SCFA, especially acetate and propionate produced by colonic fermentation of colonic bacteria, have also been associated with the insulin sensitized effects of RS^18,32^. Another mechanism associated with insulin sensitivity is to regulate systemic inflammation by altering both gut microbiota and intestinal permeability.^33^. In this meta-analysis of HOMA-S%, one trial^9^ showed the effect on inflammatory marker (hs-CRP) was not significantly changed by RS. Low-sample size and nondiabetic, including metabolic syndrome may be a cause in significant heterogeneity in analysis.

Although there were 144 diabetics in the included trials^15,20,21^, mean fasting insulin and glucose concentration at the baseline were 12.16 mIU/L and 6.98 mmol/l (diabetic: 8.31 mmol/l; nondiabetic: 6.06 mmol/l), respectively. A mild decrease was showed in the trials for analysis of fasting glucose^15,17,19,20,22,23,25,27^ and fasting insulin^15,18,19,21–23,25,27^. No significant difference was found in the trials for analysis of fasting glucose^18,24,26^ and four data showed an increase in fasting insulin^17,23,26,27^ and two in fasting glucose^23,27^, which may have prevented a significant effect on analysis of glucose with nondiabetic. Colonic fermentation of HAM-RS2 increases acetate and propionate concentration^32^. In our study, one trial^18^ showed the difference of SCFA after RS supplementation, however, there was no significance. Circulating SCFA, especially propionate, may also increase insulin secretion by binding to PPAR-γ receptors in adipose tissue^32^. The mechanism by which RS may decrease the fasting glucose has been investigated by many experimental studies, but it is considerable ambiguity. A study has shown that RS meets prebiotic criteria and can stimulate an increase of endogenous *Bifidobacteria*^34^. The increase in Clostridium cluster IV was negatively associated with fasting insulin and glucose, while a positive correlation between Propionibacterium, Bacteroides intestinalis, Bacteroides vulgates, and fasting glucose was found in another study^35^.

Some limitations of our study should be taken into consideration. First, we excluded some trials which did not provide baseline characteristic without difference. The plasma glucose and insulin were calculated as the positive area under the curve, thus, we excluded those studies for further analysis, which may influence the accuracy of the overall results. Second, in some meta-analyses, the number of studies is relatively limited, which may cause problems for evaluation of heterogeneities and publication bias and finally reduce the confidence of the results. Third, our study did not include the subjects with BMI <25, and establish the subgroup analysis according to the dosage and duration of RS. Fourth, there is a significant heterogeneity and possible publication bias in our study. Although there was no publication bias found for all the analysis, significant heterogeneity was found in fasting glucose, fasting insulin, HDL-c, HOMA-S%, HOMA-B%, and HOMA-IR, and this heterogeneity remained significant for analysis of trials with nondiabetic which depended on different countries, RS types, duration of treatment, and other unforeseen factors. Finally, the dietary intake may vary within and between individuals, which may lead to changes in insulin, glucose homeostasis, and lipid. Another important issue to consider is the composition of intestinal microflora, which is the main goal of metabolic improvement.

## Conclusion

In summary, this meta-analysis showed that RS increased HOMA-S% and reduced fasting insulin, fasting glucose, LDL-c concentration, HbA1c, and HOMA-B%, in overweight or obese adult, and they also decreased fasting glucose and HOMA-IR in overweight or obese adult with diabete. However, due to potential confounding, individual variations and gut microbiota composition, this result should be carefully considered and be confirmed by further study.
